# Caregiver Relationships and Distress Moderated by Collectivism

**DOI:** 10.1093/geroni/igaa057.1105

**Published:** 2020-12-16

**Authors:** Eunbea Kim, Mary Rogers, Erica Szkody, Cliff McKinney

**Affiliations:** 1 Mississippi State University, Gyeongsan-si, Kyongsang-bukto, Republic of Korea; 2 Mississippi State University, Mississippi State, Mississippi, United States

## Abstract

The number of older populations raising their grandchildren has increased. Past research has indicated the distress custodial grandparents’ experience is related to their family relationships (Hayslip, Shore, & Emick, 2006). Family relationships are also influenced by a variety of factors such as social history, culture, family structure, and individual differences (Uhlenberg & Kirby, 1998). The current study evaluated the influence of culture on the relationship between caregiver relationship quality and mental health by examining 885 children (18-25 years; M=18.93). This study also compared the difference in cultural impact between custodial grandparents-grandchildren and biological parents-children. Measures included the Network of Relationships Inventory, Hofstede Cultural Questionnaire, and Adult Behavior Checklist. Path analysis was conducted using AMOS 26.0 which resulted in an interaction between relationship closeness and collectivism to predict custodial grandparent depressive symptoms. Custodial grandparents who reported a lower level of closeness with their grandchildren in a higher collectivistic culture reported a significantly higher level of depression symptoms than those in a more individualistic culture, particularly for custodial grandmothers. However, custodial grandparents who reported a higher level of closeness with their grandchildren in a higher collectivistic culture reported significantly lower levels of depressive symptoms than those in a more individualistic culture. Furthermore, compared to biological parents, custodial grandparents reported significantly lower levels of depressive symptoms when reporting higher collectivistic culture. These findings will inform the need for more research to assess factors of cultural features that reduce psychological problems and support family relationships to adapt psychological therapies in older adults.

